# Ultrasound-Guided Botulinum Toxin Infiltrations in Essential Tremor Patients: A 36-week Follow Up

**DOI:** 10.5334/tohm.957

**Published:** 2025-03-03

**Authors:** Gabriel Salazar, Iolanda Caballero

**Affiliations:** 1 Department of Neurology, Consorci Sanitari de Terrassa, Barcelona, Spain

**Keywords:** essential tremor, botulinum toxin, ultrasound guidance

## Abstract

**Background:**

Essential tremor (ET) presents therapeutic challenges as oral therapies, are often partially effective and carry adverse effects. Deep Brain Stimulation and High-intensity Focused Ultrasound targeting the ventral intermediate thalamic nucleus show efficacy in managing ET; however, their cost and invasiveness deter some patients. Botulinum toxin infiltrations for ET in the upper limbs have been limited by adverse effects. Most studies used manual or electromyography guidance, while ultrasound guidance has been less explored. The purpose of the present study was to investigate the potential long-term improvement in tremor and quality of life among ET patients following ultrasound-guided IncobotulinumtoxinA (IncoBoNT) infiltrations.

**Methods:**

We present 18 ET patients who received IncoBoNT injections guided by ultrasounds. We also propose an anatomo-physiological paradigm for targeting muscles in ET patients based on two different tremor patterns.

**Results:**

Eighteen ET patients (mean age 68.2 years) were followed over 12 months. After 36 weeks, patients with supination/pronation (SPP) and flexion/extension (FEP) patterns showed significant TETRAS score improvements: 46.4% in SPP (p = 0.0022) and 48.2% in FEP (p = 0.0021). The QUEST-QOL score also improved (65% in SPP, p = 0.0018; 62.7% in FEP, p = 0.0018). All patients presented notable improvements in mean scores on the self-evaluating spiral test and neurophysiological measures (p < 0.01 for all). Treatment effects lasted 8–12 weeks, with temporary numbness and pain reported, and no cumulative effects observed.

**Discussion:**

Ultrasound-guided IncoBoNT infiltrations show promise for oral treatment-resistant ET patients with minimal adverse effects. The anatomophysiological paradigm utilized proved beneficial for our patients, although tremor pattern variability remains a consideration.

**Highlights:**

Essential tremor patients often face limited options, as oral therapies often yield only partial efficacy, and invasive interventions, like Deep Brain Stimulation, may not always be viable. In this open-label study, 18 patients received ultrasound-guided IncobotulinumtoxinA injections, showing significant tremor improvement and enhanced quality of life, with minimal adverse events reported.

## Introduction

Essential tremor (ET) is one of the most prevalent movement disorders in both adults and children, as reported by epidemiological studies conducted across various movement disorder units worldwide. ET is frequently a hereditary disorder characterized by tremors in the limbs, head, and voice. Epidemiological data indicate that up to 5% of the adult population is affected by ET, with 5–30% of adult ET cases reporting symptom onset during childhood [1,2,3,4,5,6,7]. ET considerably diminishes quality of life for most patients, affecting their daily activities and causing social challenges for both ET patients and their relatives. This impact is exacerbated by the high prevalence of adverse effects and the limited efficacy of available oral therapies [8,9].

An updated consensus statement in 2018 redefined ET as an isolated action tremor affecting both upper extremities for a minimum duration of three years. Although ET primarily involves the upper limbs, tremor can also be present in other areas, such as the neck or vocal cords. When treatment is indicated, propranolol and primidone are considered first-line therapeutic options [9,10,11]. However, for patients who exhibit insufficient response to these medications or experience adverse effects, alternative therapies may be considered, including Magnetic Resonance Guided Focused Ultrasound (MRg-FUS) VIM thalamotomy, deep brain stimulation (DBS), and botulinum toxin (BoNT) infiltrations. It is important to note MRg-FUS and DBS are more invasive and expensive treatments, while BoNT offers only partial efficacy and is associated with frequent adverse effects [9,12,13,14,15,16,17].

In this article, we present an open-label study without controlled follow-up, involving 18 patients who met the clinical criteria for ET and did not respond to conventional oral therapy. These patients experienced severe and disabling hand tremors and were treated with ultrasound-guided injections of IncobotulinumtoxinA (IncoBoNT) targeting specific forearm muscles (pronator teres/flexor carpi ulnaris/flexor carpi radialis) in one limb, according to the individual tremor pattern (either supination/pronation pattern [SPP] or flexion/extension pattern [FEP]). This ultrasound-guided method offers a promising therapeutic approach for managing ET, paving the way for further research and clinical applications.

## Methods

### Study Population

Patients enrolled in the study were required to meet the clinical criteria for ET and demonstrate resistance to at least two widely accepted oral anti-tremor medications. Additionally, these patients reported significant disability impacting their quality of life due to severe tremor. All participants signed an informed consent form, which detailed the off-label use of BoNT as a first-line therapy for tremor. The protocol was approved by the hospital’s ethical committee.

The inclusion criteria specified ET patients with severe, medication-resistant tremor (defined as lack of improvement with at least two, and in most cases three or four, conventional anti-tremor medications over several years). Furthermore, eligible patients expressed unwillingness to undergo more invasive treatments, such as MRg-FUS thalamotomy or DBS VIM-Thalamic stimulation. However, they remained open to considering MRg-FUS or DBS of the VIM nucleus as potential future options if necessary.

### Intervention

Patients received injections of BoNT type A (IncoBoNT, Merz Pharmaceuticals GmbH), administered with guidance of an ultrasound device (GE linear 12 Hz probe, MSK software with elastography).

Two distinct tremor patterns in the upper limbs were identified, based on tremor phenomenology and muscle functionality. An accelerometer was used to determine the directional orientation of the treated arm. These patterns were classified as SPP and FEP.

**Figure 1:**
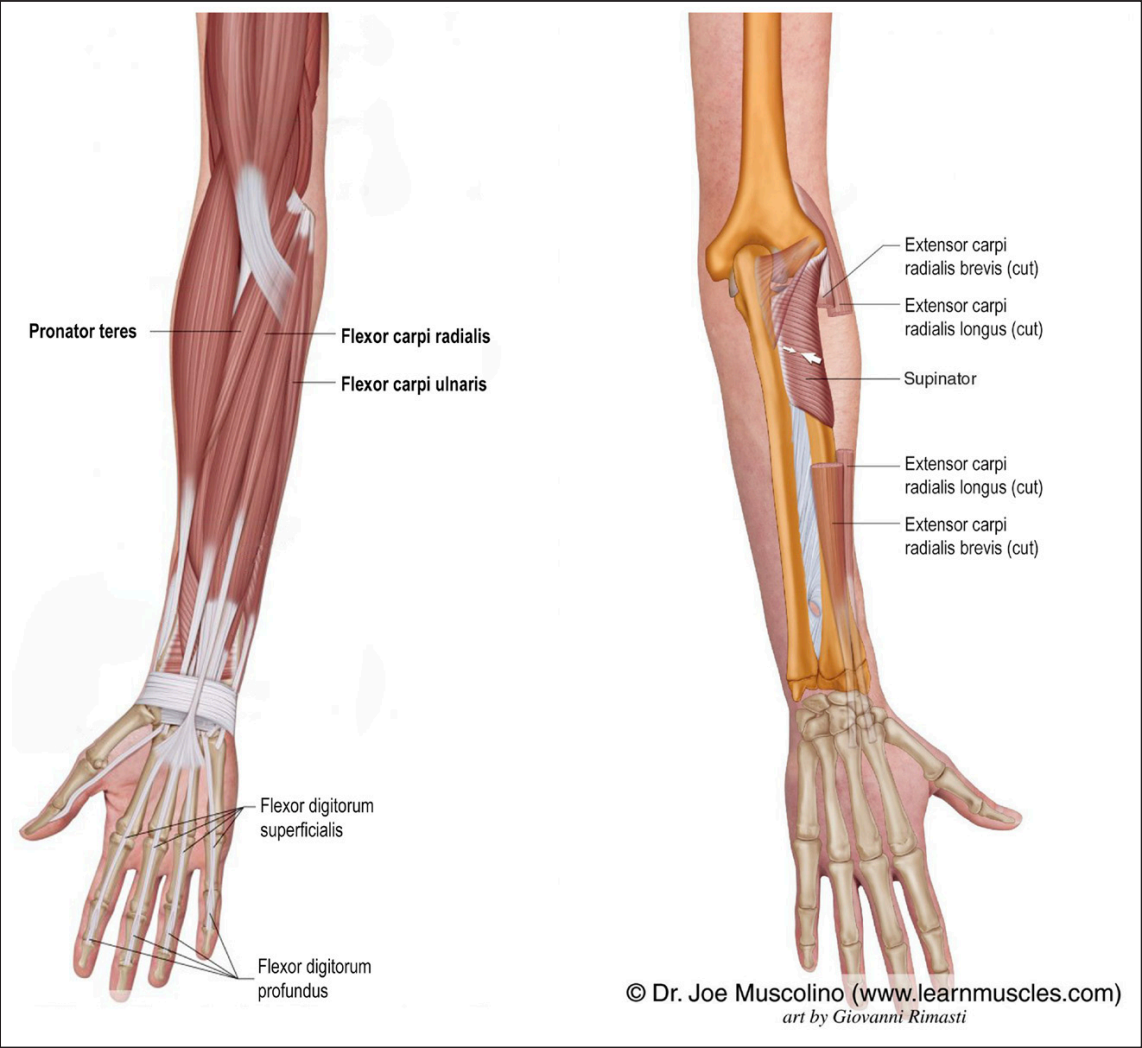
**Anatomical-functional paradigm of the pronator teres and flexor carpi muscles**. Schematic representation of the forearm and finger muscles, illustrating the muscles involved in the anatomical-functional paradigm. The muscles targeted for infiltration are highlighted in bold.

Electromyography (EMG) evaluation with surface electrodes (recording both agonist and antagonist muscles involved in each tremor pattern) and accelerometry were used alongside clinical evaluation to differentiate between the two tremor patterns. EMG surface electrodes were placed on the extensor and flexor muscles of the carpus to identify which muscle exhibited predominant overactivity (e.g., flexor carpi ulnaris, flexor carpi radialis, pronator teres). Additional electrodes were placed on the supinator and extensor muscles (Figure 1). An accelerometer was positioned on the fingers to monitor hand movement direction. The sensors facilitated the measurement of dynamic forces by detecting changes across the X, Y, and Z axes. This combined approach (clinical evaluation, identification of predominant EMG overactivity, and dynamic assessment via the accelerometer) enabled us to determine the functional pattern effectively.

Patients were asked to maintain the treated arm in three different positions to observe tremor characteristics: (1) a postural position, holding the arm at a 90-degree angle to the torso in an anterior direction; (2) an action/kinetic tremor position, simulated by bringing a bottle to the mouth as if drinking water; and (3) a resting position, with the arm resting over the same side thigh while seated. The accelerometer helped resolve uncertainties regarding the movement direction of the treated limb, such as distinguishing between extension and pronation in certain cases. In cases where a mixed pattern raised uncertainty regarding SPP or FEP, clinical criteria prevailed. Notably, the anti-gravitational muscles (such as the wrist extensors or supinator muscles) were excluded from injection targets.

Once the tremor pattern was classified, patients with an SPP received injections of 50 units (5IU/0.1 mL) of IncoBoNT into the pronator teres muscle at two specific sites (at its most proximal insertion and mid-muscle), with 25IU administered per site, carefully avoiding the flexor digitorum and flexor carpi muscles. Conversely, patients with an FEP were injected with 50IU of IncoBoNT into the flexor carpi radialis and flexor carpi ulnaris muscles (Figure 1) (one site in each muscle, 25IU per muscle, totaling 50IU), to prevent toxin diffusion to the flexor digitorum muscles, which are easily visualized via ultrasound (Video record 1). Care was taken to avoid nearby nerves and blood vessels during the procedure to minimize risks. Furthermore, all patients were evaluated using a dynamometer during each Botulinum Toxin infiltration session to measure finger strength. Grip strength was considered normal within the range of 40–50 kgf.

**Video 1:** Video demonstrating an essential tremor patient exhibiting a supination/pronation pattern, presented pre- and post-ultrasound-guided botulinum toxin infiltration.

Patients received injections every three months over a nine-month period, totaling three sessions of IncoBoNT infiltrations at fixed intervals for all participants.

The primary assessment outcomes post-infiltration were as follows: (1) tremor frequency and amplitude, measured via EMG; (2) tremor-related quality of life; and (3) monitoring of adverse effects.

Patients attended four scheduled clinical visits: (1) a baseline (initial) visit; (2) a 12-week follow-up visit; (3) a 24-week follow-up visit; and (4) a final 36-week visit follow-up visit. During each visit, the following assessments were conducted: (1) tremor severity was assessed using the TETRAS subscale specific to the treated upper limb; (2) tremor frequency and amplitude were determined using EMG on the treated limb; (3) tremor-related quality of life was assessed using the QUEST scale; (4) video recordings captured the tremor characteristics; (5) all potential adverse effects were documented.

### Data analysis

Results were evaluated after each infiltration session (12, 24, and 36weeks) by comparing data to the baseline clinical measures, aiming to avoid misinterpretation of partial results. The primary focus of this study was not assessing the cumulative effects of successive BoNT infiltrations on tremor, although this remains an interesting topic for exploration in future.

For statistical analysis, the primary assessment outcomes from the initial visit were compared to those from the final 36-week visit (final evaluation) using the chi-square test.

## Results

A total of 18 patients with ET who met the inclusion criteria were recruited and monitored over a 12-month period. Patient ages ranged from 47 to 78 years old (mean age 68.2 ± 22.2 years), with a mean disease duration of 13.5 years. The cohort comprised eight females and ten males, of whom eight reported a familial condition. All patients were severely affected, each scoring at least three points on the TETRAS scale for tremor in their most affected limb. Among the participants, eight exhibited a SPP, while ten showed a FEP (Table 1).

**Table 1:** Demographic characteristics.


N = 18	Mean (SD)

Age (years)	68.2 (22.2)

Females/Males*	8/10

Disease onset (years)	13.5 (3.1)

Total TETRAS Sub-score of the treated arm (SPP and FEP)	2.8 (0.2)

SPP	2.7 (0.4)

FEP	2.9 (0.3)

Total Self-evaluating spiral written test (0–4) SPP and FEP	2.8 (0.4)

SPP	3.1 (0.2)

FEP	2.7 (0.3)

QUEST-QOL score	45.0 (4.0)

SPP	46.0 (2.0)

FEP	43.0 (1.0)

Tremor pattern (n)	

SPP*	8

FEP*	10

Upper limb tremor*	18

Lower limb tremor*	8

Voice tremor*	6

Head tremor*	3

Resting tremor*	6


FEP, flexor/extensor pattern; QUEST-QOL, quality of life in essential tremor questionnaire; SPP, supination/pronation pattern; TETRAS, the essential tremor rating assessment scale. *Absolute frequency.

Patients scored an average of 27.0 ± 2.0 points on the physical subscale and 18.0 ± 1.0 points on the psychosocial subscale of the QUEST quality of life (QUEST-QOL) questionnaire, resulting in a total average score of 45.0 ± 4.0 (Table 1).

At the 36-week evaluation, SPP-ET patients demonstrated a mean TETRAS score of 1.5 ± 0.4 points for tremor, representing an improvement of 46.4% (p = 0.0022). Their mean QUEST-QOL score was 18.0 ± 1.0 (physical = 7.0 ± 0.4, psychosocial = 11.0 ± 0.4), showing a 65% improvement (p = 0.0018). In the self-evaluating spiral-written test, SPP-ET patients scored an average of 1.6 ± 0.3, corresponding to a 57.8% improvement (p = 0.0021).

For patients with FEP-ET, the mean TETRAS score was 1.4 ± 0.3 points for tremor, indicating an improvement of 48.2% (p = 0.0021). Their mean QUEST-QOL score was 16.4 ± 1.0 (physical = 5.0 ± 0.3.0, psychosocial = 11.4 ± 0.1), showing an improvement of 62.7% (p = 0.0018). In the self-evaluating spiral-written test, FEP-ET patients achieved an average score of 1.2 ± 0.4, making a 55.1% improvement (p = 0.002) (Table 2).

**Table 2:** 36-week follow-up after IncoBoNT ultrasound-guided infiltrations.


	BASAL, MEAN (SD)	36-WEEK, MEAN (SD)	P

SPP-ET patients			

TETRAS (sub-score for the treated arm)	2.7 (0.4)	1.5 (0.4)	0.0022

QUEST-QOL score [Ph/Psy]	46.0 (1.0) [(29.0 (0.8)/16.0 (0.4)]	16.0 (3.0) [15.0 (0.1)/1.0 (0.7)]	0.0018

Self-evaluating spiral written test (0–4)	3.1 (0.2)	1.3 (0.5)	0.0022

Neurophysiological parameters (Hz/mm)			

Postural	9.3 (1.5)/16 (2,5)	2.2 (0.3)/2 (0,6)	0.0011/0.0018

Action	11.2 (1.6)/15 (2,5)	2.5 (0.8)/1,8 (0,9)	0.0012/0.0016

Resting	7.3 (0.5)/11 (2,5)	2.3 (0.6)/1,9 (0,8)	0.0021/0.0017

FEP-ET patients			

TETRAS (sub-score for the treated arm)	2.9 (0.3)	1.4 (0.3)	0.0021

QUEST-QOL score (Ph/Psy)	43.0 (1.0) [28.0 (0.1)/15.0 (0.1)]	16.4 (1.0) [5.0 (0.3)/11.4 (0.1)]	0.0018

Self-evaluating spiral written test (0–4)	2.7 (0.3)	1.2 (0.4)	0.0022

Neurophysiological parameters (Hz)/mm			

Postural	10.2 (1.2)/17 (2,5)	1.9 (0.2)/2 (0,7)	0.0011/0.0018

Action	11.5 (2.1)/18 (2,3)	2.2 (0.7)/2 (0.8)	0.0021/0.0015

Resting	6.8 (0.4)/12 (1,6)	2.6 (0.4)/1 (0.7)	0.0019/0.0018


ET, essential tremor; FEP, flexor/extensor pattern; Ph, physical subscale; Psy, psychological subscale; QUEST-QOL, quality of life in essential tremor questionnaire; SPP, supination/pronation pattern; TETRAS, the essential tremor rating assessment scale.

Regarding the neurophysiological parameters at the 36-week evaluation, the SPP-ET patients presented a mean frequency of postural tremor of 2.2 ± 0.3 Hz, representing a 76.3% improvement (p = 0.0011), with a mean amplitude of 2 mm (SD 0, 6, p = 0.0018). For kinetic tremor, the mean frequency was 2.5 ± 0.8 Hz, showing a 77.6% improvement (p = 0.0012), with an amplitude of 1.8 mm (SD 0.9, p = 0.0016). The resting tremor frequency was 2.3 ± 0.6 Hz, an improvement of 68.4% (p = 0.0021), with an amplitude of 1,9 mm (SD 0.8, p = 0.0017).

In contrast, FEP-ET patients had a mean frequency of postural tremor of 1.9 ± 0.2 Hz, reflecting an 81.3% improvement (p = 0.0011). The mean frequency of kinetic tremor was 2,2 ± 0,7 Hz, with an 80.8% improvement (p = 0.0021), and the resting tremor frequency was 2.6 ± 0.4 Hz, an improvement of 61.7% (p = 0.0019). For amplitude, FEP-ET patients exhibited values of 2 mm for action tremor (p = 0.0018), 1 mm for kinetic tremor (p = 0.0015), and 1 mm for resting tremor (p = 0.0018) (Table 2).

BoNT begins to take effect within 24–72 hours, as it disrupts the synaptosomal process, with full effects occasionally requiring up to five days, and the therapeutic effects of the toxin typically persisting for 8–12 weeks. No evidence of a carry-over (accumulative) effect from subsequent infiltrations was observed during this follow-up period. A prospective study to evaluate potential progressive and cumulative effects of BoNT after multiple treatment cycles could be valuable, although this was beyond the scope of the present study.

Adverse effects were reported by some patients. Among SPP-ET patients, two reported numbness and mild weakness in the first and second fingers of the treated arm, and three experienced mild to moderate, short-lasting pain at the infiltration site. In the FEP-ET group, three patients noted mild, short-lasting weakness in the first and second fingers of the treated limb. Grip strength was assessed, with a mean baseline value of 45 kgf (range: 40–55). Among the two SPSP-ET patients who experienced mild finger weakness, one had a grip strength of 45 kgf at baseline, which decreased to 42 kgf after BoNT infiltration. The other patient had a baseline grip strength of 50 kgf, which decreased to 45 kgf post-infiltration. Regarding the three FEP-ET patients who reported mild, short-lasting weakness after BoNT infiltration, one patient showed a grip strength of 50 kgf at baseline, decreasing to 46 kgf on two occasions; another patient had a baseline grip strength of 48 kgf, decreasing to 46 kgf once; and the third patient had a baseline grip strength of 52 kgf, decreasing to 48 kgf once. All the adverse effects were temporally related to the most recent injection cycle and were of limited duration.

## Discussion

Despite the advancements in invasive surgical therapies such as DBS and less invasive options like MRg-FUS, ET patients continue to face significant challenges [6,18]. These procedures remain inaccessible to the majority of patients worldwide and come with limitations, including age restrictions, brain atrophy, cognitive decline, and the presence of calcium deposits in the skull for MRg-FUS [19,20,21].

BoNT has shown efficacy in treating some hyperkinetic movement disorders over the last few decades. Medical reports have documented that BoNT can reduce the intensity of postural and action tremors in ET patients [22,23]. However, these improvements in tremor symptoms do not consistently lead to enhanced quality of life, as adverse effects (like finger weakness and clumsiness in handling objects) can adversely impact daily activities [24]. Consequently, many neurologists have opted to discontinue this therapy as a first-line treatment for tremor relief. We believe that the lack of a reliable visualization tool to accurately guide BoNT injections into tremor-involved muscles has been a major limitation, affecting both efficacy and the high rate of adverse effects reported [25]. The widespread diffusion of the toxin into both deep and superficial finger flexors has likely contributed to the weakness experienced by these patients historically.

Therefore, ultrasound guidance for BoNT injections has emerged as a promising technique for treating various movement disorders. Some studies reported improved efficacy and reduced adverse effects with ultrasound guidance [26,27]. A Bayesian network meta-analysis suggested ultrasound guidance may be superior to other techniques for limb spasticity treatment [28]. Although BoNT diffusion cannot be completely avoided due to its properties [29], this technique offers real-time imaging of target structures, enabling precise delivery of BoNT into the specific muscles involved in tremor, while avoiding non-target muscles and minimizing the risk of injuring critical anatomical structures, such as nerves and blood vessels, which may occur during unguided BoNT infiltrations [27]. For instance, Henzel et al. (2010) assessed 18 patients with upper extremity flexor spasticity and reported that ultrasound localization provides several advantages, including more precise muscle targeting (particularly in complex anatomical regions), the ability to ensure that the injectate remains within fascial borders, avoidance of neurovascular structures, and reduced pain due to minimized reliance on blind probing compared to landmark-based techniques [30]. Particularly in sensitive populations, such as adolescents with ET who are highly sensitive to pain, ultrasound-guided BoNT injections offer a distinct advantage by significantly reducing procedural discomfort. EMG-guided injections, by contrast, often require multiple needle insertions—both for recording muscle activity and delivering the toxin—which can be particularly distressing for young patients. Ultrasound, on the other hand, enables real-time visualization of the target muscle, eliminating the need for additional needle passes. This reduction in pain not only enhances the overall patient experience but also improves compliance with treatment, making ultrasound-guided injections an ideal approach for managing ET in this population. However, ultrasound-guided injections have not been widely adopted across clinical centers. One barrier is the requirement for specific training and expertise [31], which may not be readily available in settings without a strong focus on neuromuscular disorders. Additionally, implementing ultrasound-guided techniques involves initial costs for equipment acquisition and maintenance, which some centers may find prohibitive. These challenges may hinder the integration of ultrasound-guided injections into routine clinical practice.

This 36-week follow-up study describes a cohort of 18 patients with ET who experienced substantial impairment in quality of life and demonstrated resistance to several oral drug therapies. Injections were conducted with ultrasound guidance, following an anatomical and physiological paradigm tailored to limb orientation. This paradigm proposed in the present study has enabled more precise BoNT injections, thereby improving efficacy in targeting the muscles directly involved in the tremor. The study documents a significant and consistent reduction in tremor severity following the administration of IncoBoNT injections into targeted forearm muscles. We observed a clear improvement in neurophysiological parameters in all patients, as well as across all types of tremors (resting, kinetic, and postural). The reduction in frequency and amplitude was evident when comparing EMG parameters from baseline to 36 weeks. Previous studies have reported improvements in tremor amplitude (cervical and limb) but only mild improvements in tremor frequency. We currently lack an explanation for this discrepancy in tremor improvement. However, other authors have reported improvements in both amplitude and frequency in voice tremor after BoNT infiltration, similar to those observed in our patients [32].

It is well established that BoNT typically induces a reduction in the strength of the infiltrated muscle [12]. In hyperactive muscles like the pronator teres and flexor carpi muscles, this reduction in strength can effectively decrease the frequency and amplitude of tremors in the infiltrated arm without affecting more functional muscles, such as the finger flexors. It is possible that repeated BoNT infiltrations could cause a carry-over effect, potentially leading to a more persistent tremor reduction for ET patients. However, this remains speculative, as the present study was not designed to evaluate the long-term effects of BoNT infiltration in ET patients.

In terms of adverse effects, our patients showed a relatively low incidence of mild adverse effects, such as mild finger weakness and localized infiltration pain, all directly associated with the most recent BoNT infiltration cycle. We attribute this low incidence of adverse effects to the use of ultrasound as a guidance tool, which enabled precise targeting of BoNT administration. By avoiding the finger flexor muscles and instead releasing the toxin into the pronator teres or flexors of carpi muscles (located at a distance from the finger flexor muscles previously linked to finger weakness) we minimized unintended impacts on finger function. However, further investigation using ultrasound-guided BoNT injections is needed to fully understand the side effect profile at the doses used in this study.

Nevertheless, this study presents several limitations that must be acknowledged. First, the absence of a placebo-controlled group restricts the ability to draw definitive conclusions about the intervention’s efficacy, as it leaves open the possibility that observed improvements may be influenced by placebo effects. Although consistent improvements across a minimum of three sessions suggest an effect beyond placebo, future studies incorporating placebo controls will be essential for more robust conclusions. Additionally, while this study provides valuable insights into the long-term management of ET with botulinum toxin, the fixed-dose, single-muscle injection approach could be reconsidered in favor of a more individualized strategy. Finally, future studies incorporating short-term follow-up at 4–8 weeks could enhance the clinical relevance of the findings and strengthen empirical support for ultrasound’s superiority as a guidance method.

## Conclusions

We believe that the precise, systematic, and ultrasound-guided infiltration of BoNT into the forearm muscles of ET patients can significantly reduce tremor intensity and frequency. This clinical follow-up demonstrates a positive impact on our patients’ quality of life. However, we recognize the need for further studies to substantiate the scientific evidence supporting ultrasound-guided BoNT administration in ET patients and to optimize strategies for enhancing their quality of life.
